# The Role of Autophagy in Copper-Induced Apoptosis and Developmental Neurotoxicity in SH-SY5Y Cells

**DOI:** 10.3390/toxics13010063

**Published:** 2025-01-17

**Authors:** Lu Lu, Ying Zhang, Wei Shi, Qian Zhou, Zhuoqi Lai, Yuepu Pu, Lihong Yin

**Affiliations:** Key Laboratory of Environmental Medicine Engineering, Ministry of Education, School of Public Health, Southeast University, Nanjing 210009, China; 230229018@seu.edu.cn (L.L.); 101300315@seu.edu.cn (Y.Z.); 230239085@seu.edu.cn (W.S.); 230229470@seu.edu.cn (Q.Z.); 220223707@seu.edu.cn (Z.L.); yppu@seu.edu.cn (Y.P.)

**Keywords:** copper, developmental neurotoxicity, autophagy, Wnt signaling pathway

## Abstract

Copper (Cu) is a global environmental pollutant that poses a serious threat to humans and ecosystems. Copper induces developmental neurotoxicity, but the underlying molecular mechanisms are unknown. Neurons are nonrenewable, and they are unable to mitigate the excessive accumulation of pathological proteins and organelles in cells, which can be ameliorated by autophagic degradation. In this study, we established an in vitro model of Cu^2+^-exposed (0, 15, 30, 60 and 120 μM) SH-SY5Y cells to explore the role of autophagy in copper-induced developmental neurotoxicity. The results showed that copper resulted in the reduction and shortening of neural synapses in differentiated cultured SH-SY5Y cells, a downregulated Wnt signaling pathway, and nuclear translocation of β-catenin. Exposure to Cu^2+^ increased autophagosome accumulation and autophagic flux blockage in terms of increased sequestosome 1 (p62/SQSTM1) and microtubule-associated protein 1 light chain 3B (LC3B) II/LC3BI expressions and inhibition of the phosphatidylinositol 3-kinase (PI3K)/Akt/mTOR pathway. Furthermore, copper induced apoptosis, characterized by increased expressions of Bcl2 X protein (Bax), caspase 3, and Poly (ADP-ribose) polymerase (PARP) and decreased expression of B-cell lymphoma 2 (Bcl2). Compared with the 120 μM Cu^2+^ exposure group alone, autophagy activator rapamycin pretreatment increased expression of Wnt and β-catenin nuclear translocation, decreased expression of LC3BII/LC3BI and p62, as well as upregulated expression of Bcl2 and downregulated expressions of caspase 3 and PARP. In contrast, after autophagy inhibitor chloroquine pretreatment, expressions of Wnt and β-catenin nuclear translocation were decreased, expression levels of LC3BII/LC3BI and p62 were upregulated, expression of Bcl2 was decreased, while expression levels of caspase 3, Bax, and PARP were increased. In conclusion, the study demonstrated that autophagosome accumulation and autophagic flux blockage were associated with copper-induced developmental neurotoxicity via the Wnt signaling pathway, which might deepen the understanding of the developmental neurotoxicity mechanism of environmental copper exposure.

## 1. Introduction

Due to the extremely widespread use of copper, the health impacts of its environmental pollution are of concern [1]. Copper is an indispensable metal element in the physiological state and plays a vital part in lots of physiological processes, such as mitochondrial respiration, scavenging of reactive oxygen species, and synthesis of neurotransmitters [2,3]. However, the widespread use of Cu-containing fungicides and pesticides and Cu mining, leading to dramatic increases in environmental copper levels [4,5]. Excessive copper is hazardous for the health of living organisms, including humans, through bioaccumulation effects [6]. Excessive copper may accumulate in the liver, kidneys, and neurological system, associated with the development of neurodegenerative diseases, such as Alzheimer’s disease, Huntington’s disease, and Parkinson’s disease [7,8]. The developing brain is more susceptible to damage from environmental toxins than the adult brain [9]. Epidemiological studies have found that high copper caused changes in basal ganglia structures and hypokinesia, leading to autism spectrum disorder (ASD) and attention-deficit hyperactivity disorder (ADHD) in 8 to 16-year-old children [10,11]. The maternal copper level was negatively correlated with neuropsychological development of the offspring at 12 months [12]. Animal studies indicated that copper induced hippocampal tissue damages and synaptic dysfunction, causing motor function injury and memory impairment [13,14]. Nevertheless, the detailed mechanisms of Cu-induced developmental neurotoxicity remain largely obscure.

Among the signaling pathways regulating neural development, the Wnt signaling pathway is essential, regulating the proliferation and differentiation of stem cells and progenitor cells [15]. The most characterized of Wnt signaling pathway is the Wnt/β-catenin signaling pathway [16]. When Wnt signaling is on, stabilized β-catenin accumulates in the cytoplasm and is further translocated to the nucleus, regulating the expressions of target genes [17]. Conversely, β-catenin is degraded by the proteasome through ubiquitination modification [18]. The transcripts of canonical Wnt/β-catenin signaling proteins and developmental transcription factors were downregulated by copper nanoparticles, causing the inhibition of neuronal regeneration in rainbow trout olfactory mucosa [19]. Copper affected the survival and development of fish at an early stage of life through inhibiting antioxidant enzymes and the Wnt/β-catenin signaling pathway [20]. This study speculated that the developmental neurotoxicity induced by Cu^2+^ could be correlated with the Wnt signaling pathway. However, the understanding on the position of the Wnt pathway in copper-induced developmental neurotoxicity is still very limited.

Compelling evidence showed that the mechanisms of Cu-induced neurological damage included disturbances in neurotransmitters, apoptosis, DNA double-strand breaks, oxidative stress, cuproptosis, and inflammation [21,22,23]. Due to the non-renewable nature of neurons, they are unable to alleviate the excessive accumulation of pathologic proteins and organelles in cells [9], and this can be ameliorated by autophagic degradation [24]. Autophagy, also known as autophagic flux, has been visualized as “self-eating” and plays an essential role in structural reorganization of neuronal circuits through dendritic spine formation and pruning, axonal growth, synaptic assembly, and vesicle turnover [25]. Its dynamic processes include the formation and maturation of autophagosomes, fusion of autophagosomes with lysosomes, and degradation in lysosomes [26,27]. Excessive intake of Cu disrupted mitochondrial metabolism by inhibiting mitophagy and increasing autophagosome accumulation, inducing cognitive dysfunction in Sprague-Dawley rats [28]. The current study has shown that copper treatment induced abnormal autophagy and apoptosis, leading to cell death [29]. Copper elevated LC3II and p62 protein levels, impaired autophagic flux, and induced apoptosis [21,30]. Cu induced autophagy and mitochondria-associated endoplasmic reticulum membrane dysfunction in duck renal tubular epithelial cells [31]. Excessive intake of Cu could induce oxidative stress and autophagy in the hypothalamus of broilers, which was indicated by the increased expression levels of Beclin 1 and LC3II/LC3I [32]. However, the potential link between autophagic impairment and copper-induced developmental neurotoxicity remains unclear.

This study aims to explore the function and mechanism of autophagy in developmental neurotoxicity induced by copper. SH-SY5Y is a widely used cell model for studying developmental neurotoxicity, as well as the toxic effects generated by environmental contaminants on various functional neural cells, because it could be differentiated by all-trans retinoic acid (RA) and shares functional and biochemical properties of neurons and expresses neuron-specific markers [33,34,35]. An in vitro model of Cu^2+^-treated SH-SY5Y cells is established to explore whether copper causes developmental neurotoxicity by inducing autophagic impairment.

## 2. Material and Methods

### 2.1. Chemicals and Reagents

Copper sulfate pentahydrate (CuSO_4_·5H_2_O, molecular weight 249.69, CAS No. 7758-99-8, purity > 98%) was from Sigma (Livonia, MI, USA), rapamycin (Rapa; chemical formula C_51_H_79_NO_13_, molecular weight 914.18, CAS No. 53123-88-9, purity > 99%) was from Selleck (Houston, TX, USA), chloroquine (CQ; chemical formula C_18_H_26_ClN_3_, molecular weight 319.87, CAS No. 54-05-7, purity > 99%) was from MedChemExpress (Princeton, NJ, USA), and retinoic acid (RA; chemical formula C_20_H_28_O_2_, molecular weight 300.44, CAS No. 302-79-4, purity ≥ 98%) was from Sigma-Aldrich (Livonia, MI, USA). The Annexin V-Fluoresceine isothiocyanate (FITC)/propidium iodide (PI) apoptosis detection kit was from Meilunbio (Dalian, China). NE-PER nuclear and cytoplasmic extraction reagents were from Thermo Fisher Scientific (Waltham, MA, USA). Cell Counting Kit-8 was from Beyotime Biotechnology (Shanghai, China). The following antibodies were used: anti-βIII-tubulin (M0805-8), Wnt1 (ER65317), Wnt5a (ET1706-33), glycogen synthase kinase-3 (GSK3β, ET1607-71), p-GSK3β (ET1607-60), p62(HA721171), and Atg7 (ET1610-53), from Huabio Biotechnology (Hangzhou, China); anti-β-catenin (51067-2-AP) and PARP (13371-1-AP), from Proteintech (Wuhan, China); caspase 3 (A19654), Bax (A0207), p-PI3K (AP0427), β-actin (AC026), and H3 (A2348), from ABclonal Technology (Wuhan, China); anti-Bcl2 (15071), PI3K (4294), Akt (9272), p-Akt (9271), mTOR (2983), and p-mTOR (2974), from Cell Signaling Technology (Boston, MA, USA); anti-LC3B (EPR18709), from Abcam (Cambridge, MA, USA). Horseradish peroxidase (HRP)-conjugated secondary antibodies were from ABclonal Technology (Wuhan, China). Alexa Fluor^®^ 594-conjugated goat anti-mouse IgG (H+L) was from Servicebio (Wuhan, China). Alexa Fluor^®^ 555-conjugated goat anti-mouse IgG polyclonal antibody (HA1118) was from Huabio Biotechnology (Hangzhou, China).

### 2.2. Cell Culture and Treatment

The human neuroblastoma SH-SY5Y cell line was from the Key Laboratory of Environmental Medicine Engineering, Ministry of Education, School of Public Health, Southeast University. The cells were cultured in MEM/F12 medium (Procell, Wuhan, China) with 15% fetal bovine serum (FBS; Excell Bio, Shanghai, China) and 1% penicillin and streptomycin (Gibco, Carlsbad, CA, USA) at 37 °C under humidified atmospheric conditions containing 5% CO_2_. CuSO_4_·5H_2_O was diluted in pure water (40 mM) and kept at 4 °C until it was added to complete medium. Guidelines for drinking water quality issued by the World Health Organization (WHO, fourth edition, Geneva, Switzerland), the US Environmental Protection Agency (EPA), and China (GB 5749-2006, Beijing, China) stipulate the guideline values of Cu as 2.0 mg/L [36], 1.3 mg/L [37], and 1.0 mg/L [38], respectively, and in combination with cell viability assays, SH-SY5Y cells were exposed to 0, 15, 30, 60, and 120 μM Cu^2+^ for 48 h. According to previous studies, Rapa (200 nM) was pretreated with cells for 2 h and CQ (10 μM) was pretreated with cells for 1 h before Cu^2+^ exposure [39,40].

### 2.3. Cell Viability Assay

CCK-8 (Beyotime Biotechnology, Shanghai, China) was used to detect the cell viability after 12, 24, and 48 h of 0–120 μM Cu^2+^ treatment. Firstly, SH-SY5Y cells were treated with copper in a 96-well plate (1 × 10^4^ cells/well). Then, CCK-8 solution and cell culture medium were added into the 96-well plate (1:10, *v*/*v*). The 96-well plate was incubated at 37 °C for 90 min and the absorbance at 450 nm was measured by a microplate reader (Biotek, Winooski, VT, USA).

### 2.4. Evaluation of Neurite Outgrowth

Firstly, SH-SY5Y cells were treated with Cu^2+^ with/without Rapa or CQ for 48 h in a 6-well plate (1 × 10^5^ cells/well). Then, the medium was replaced with MEM/F12 medium containing 1% FBS and 10 μM RA. Every 48 h, the medium was renewed. After 5 days of incubation, 3 pictures of the different Cu^2+^ concentrations were randomly taken under a microscope (Olympus, Tokyo, Japan). All differentiated SH-SY5Y cells displayed in the pictures were counted, and the total number of branches of all visible neurites in the pictures was measured using ImageJ 1.52 software. A sufficient number of fields of view were obtained from approximately 1000 cells for image analysis.

### 2.5. Immunofluorescence

The SH-SY5Y cells were inoculated onto coverslips in a 6-well plate. After the indicated treatments, cells were fixed in 4% paraformaldehyde for 20 min at room temperature and then blocked in 5% bovine serum albumin (BSA) for 1 h after being permeabilized with 0.2% Triton X-100 at room temperature. Then, coverslips were incubated with primary antibodies (1:100) at 4 °C overnight. Coverslips were washed with phosphate-buffered saline (PBS) 3 times and incubated with fluorescent secondary antibodies at room temperature for 1 h. DAPI (Beyotime Biotechnology, Shanghai, China) was used to stain the nucleus. Coverslips were placed onto microscope slides and visualized with a fluorescence microscope (Zeiss, Oberkochen, Germany).

### 2.6. Flow Cytometry

The Annexin V-FITC/PI apoptosis detection kit was from Meilunbio (Dalian, China). After digestion of the cells with EDTA-free trypsin, the cells were gently blown off the 6-well plate and collected by centrifugation; then, the cells were washed 2 times and incubated with 100 μL of loading buffer. Next, 5 μL of Annexin V-FITC and 5 μL of PI were added and reacted for 15 min, avoiding light. The samples were assayed by a flux cytometry system (Becton Dickinson Biosciences, Milpitas, CA, USA).

### 2.7. Mitochondrial Membrane Potential (MMP, Δψm) Assay

MMP was detected with the JC-1 probe (Beyotime, Shanghai, China). JC-1 staining shows healthy mitochondria by emitting red fluorescence, whereas unhealthy cells show decreased red fluorescence and increased green fluorescence, which represents the remaining monomers in the cytoplasm. Therefore, the ratio of red/green serves as an indicator of loss of MMP. SH-SY5Y cells (2 × 10^5^ cells/well) were seeded into 6-well plates and treated with Cu^2+^ for 48 h; then, cells were washed with PBS buffer and stained with 10 μg/mL of JC-1 dye for 30 min at 37 °C, avoiding light. After washing with PBS three times, the MMP was detected by a fluorescence microscope (Zeiss, Oberkochen, Germany). The red fluorescence intensity (polymerized form of JC-1) was measured at an Ex/Em of 585/590 nm, and the green fluorescence intensity (monomerized form of JC-1) was measured at an Ex/Em of 514/529 nm.

### 2.8. Extraction of Nuclear and Cytoplasmic Protein

Nuclear and cytoplasmic extraction reagents (Thermo Fisher Scientific, USA) were operated according to the manufacturer’s instructions. Briefly, the cells were collected and washed with PBS three times. CER I was added, vortexed vigorously, and reacted on ice for 10 min and, following this, CER II was added, vortexed vigorously twice, and reacted on ice for 10 min, then centrifuged at 16,000× *g* for 5 min. The supernatant was the cytoplasmic extract. Next, NER was added to the cell precipitate, vortexed vigorously, and reacted on ice for 10 min, 4 times, and then centrifuged at 16,000× *g* for 10 min to obtain the cytoplasmic extract. The extracts were stored at −80 °C (CER I:CER II:NER = 200:11:100).

### 2.9. RNA Extraction and Reverse Transcription-Quantitative PCR Assays

Total RNA of SH-SY5Y cells was extracted with RNA-easy Isolation Reagent (Vazyme, Nanjing, China). Then, it was reverse transcribed to cDNA using the PrimeScript™ RT Reagent Kit (Takara, Osaka, Japan). Real-time PCR was performed using StepOnePlus (ABI, Los Angeles, CA, USA) with a total volume of 10 μL, containing the SYBR Green real-time PCR Master mixture (TAKARA, Osaka, Japan). Primer sequences used in the study were as follow: LC3B: Forward: CGAACAAAGAGTAGAAGATGTCCG and Reverse: TGAGCTGTAAGCGCCTTCTA; p62: Forward: TAGGAACCCGCTACAAGTGC and Reverse: GGACCCATTTCCCATCCTGG. The PCR conditions were as follow: initial denaturant at 95 °C for 30 s, followed by 40 cycles of denaturant at 95 °C for 5 s and annealing at 60 °C for 30 s. The 2^−ΔΔCt^ method was used to analyze the gene expression levels and the β-actin was used as an endogenous control.

### 2.10. Western Blot

Total protein was lysed with radio immunoprecipitation assay (RIPA), phenylmethanesulfonylfluoride, and phosphatase inhibitor (100:1:1, Beyotime Biotechnology, China) on ice for 30 min. Total protein concentrations were quantified by the BCA kit (Thermo Fisher Scientific, USA). The protein was denatured in boiling water for 5 min and stored at −20 °C. Then, 20–60 μg protein samples were transferred onto polyvinylidene fluoride (PVDF) membranes (Millipore, Temecula, CA, USA) and incubated with primary antibodies: anti-Wnt5a (1:1000), GSK3β (1:1000), p-GSK3β (1:1000), cleaved PARP (1:500), p62 (1:5000), LC3B (1:1000), Atg7 (1:1000), β-catenin (1:5000), PARP (1:1000), caspase 3 (1:1000), Bax (1:1000), p-PI3K (1:500), β-actin (1:50,000), H3 (1:6000), Bcl2 (1:1000), PI3K (1:1000), Akt (1:1000), p-Akt (1:1000), mTOR (1:1000), and p-mTOR (1:1000), at 4 °C overnight. The bands were incubated with Horseradish peroxidase (HRP)-conjugated secondary antibodies (1:5000) after being washed with TBST. The protein expression levels were analyzed using ImageJ software, with anti-β-actin (1:50,000) as the control.

### 2.11. Statistical Analysis

GraphPad Prism 8 and SPSS 23 software were used for data analysis. The statistics were described as mean ± SD. One-way ANOVA was used to analyze the differences among multiple groups, while Dunnett’s test or Bonferroni’s post hoc test was used to analyze differences between copper treatment groups and the control group. The significance threshold was set at *p* < 0.05 and *p* < 0.01. Data are representative of at least three independent experiments and biological replicates.

## 3. Results

### 3.1. Cu^2+^ Inhibited Proliferative Activity and Differentiation Capacity of SH-SY5Y Cells

**Figure 1:**
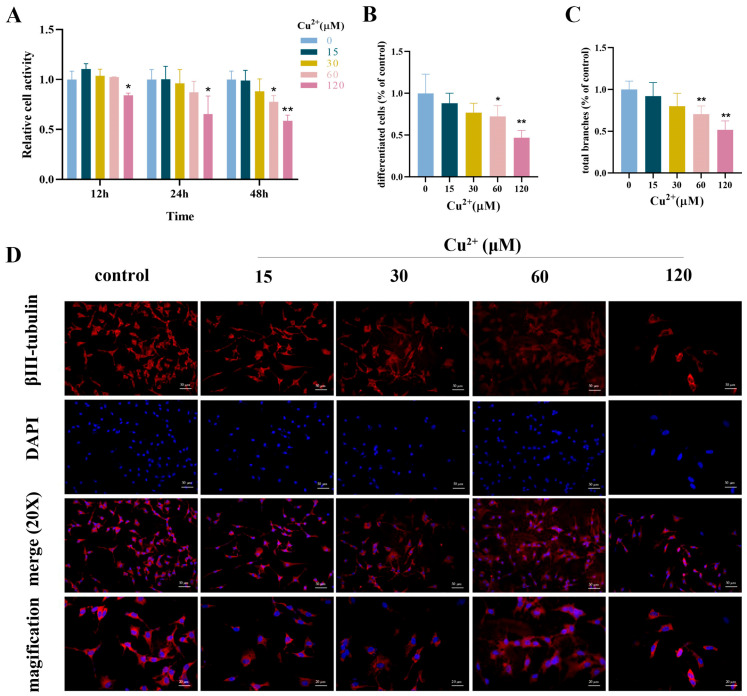
The effects of Cu^2+^ exposure on the proliferative activity and differentiation capacity of SH-SY5Y cells. (**A**) The relative cell activity of SH-SY5Y cells after exposure to Cu^2+^ (0, 15, 30, 60, and 120 μM) at different times (12, 24, and 48 h). (**B**) The proportions of differentiated cells after Cu^2+^ exposure for 48 h. (**C**) The proportions of total branches of differentiated SH-SY5Y cells after Cu^2+^ exposure for 48 h. (**D**) The immunostaining of βIII-tubulin of differentiated SH-SY5Y cells after Cu^2+^ exposure (scale bar-merge: 50 μm; magnification: 20 μm). (* *p* < 0.05 and ** *p* < 0.01. The asterisks stand for the difference between Cu^2+^ treated group and the control group).

The proliferative activity of SH-SY5Y cells was detected by the CCK-8 assay after Cu^2+^ exposure (0, 15, 30, 60 and 120 μM) at different times (12, 24, and 48 h). The activity of SH-SY5Y cells decreased with the increase in the Cu^2+^ concentration and exposure time (Figure 1A). Cu^2+^ inhibited cell proliferative activity in a concentration-dependent manner at 48 h of treatment. After Cu^2+^ exposure for 48 h, the relative cell activity of SH-SY5Y cells in the 15, 30, 60 and 120 μM Cu^2+^ groups was 98%, 88%, 77% and 58%, compared with the control group (reference, cell activity = 100%). To further evaluate the neurotoxic effects of copper, the neurite outgrowth was measured after differentiation for 5 days in RA and continuous Cu^2+^ exposure for 48 h. Cu^2+^ inhibited outgrowth of neurite in a dose-dependent manner, especially in the 60 μM and 120 μM Cu^2+^ groups. Compared with the control group, the inhibition rates of differentiated cells were 12%, 23%, 28% and 53% in Cu^2+^ exposure groups (Figure 1B), and the total branches of Cu^2+^-treated groups were 92%, 80%, 70% and 52%, respectively (Figure 1C). The results of the microscopic photographs revealed that the cells showed obvious morphological changes, such as short neurites and few branches (Figure 1D and Figure S1).

### 3.2. Cu^2^ Induced Apoptosis in SH-SY5Y Cells

**Figure 2:**
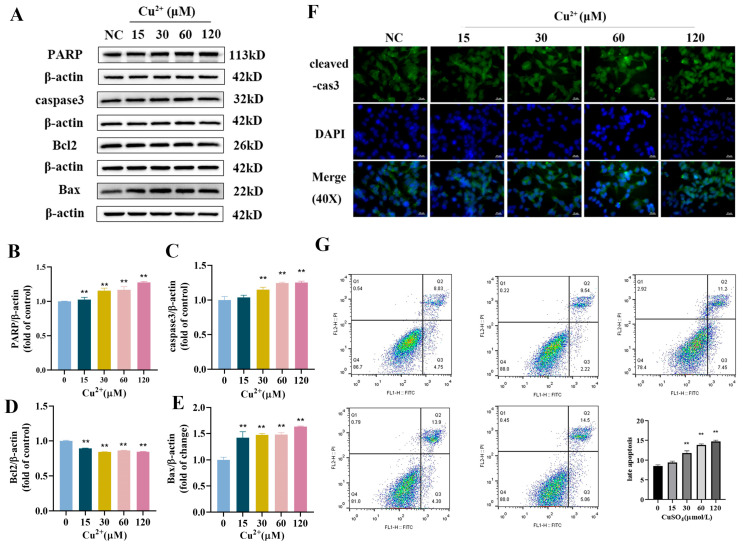
Cu^2+^ induced apoptosis in SH-SY5Y cells. (**A**) Western blot results of PARP, caspase 3, Bcl2 and Bax. (**B**–**E**) Quantitative analysis for protein expressions of PARP, caspase 3, Bcl2 and Bax. (**F**) The immunostaining of cleaved-caspase 3 of SH-SY5Y cells after Cu^2+^ treatment for 48 h (scale bar = 20 μm). (**G**) Flow cytometry of SH-SY5Y cells after Cu^2+^ treatment for 48 h. ** *p* < 0.01.

The results of the Western blot indicated that the protein expression levels of PARP, caspase 3, and Bax in Cu^2+^-treated groups were dramatically increased, while the protein expression of Bcl2 decreased with higher copper concentrations (Figure 2A–E). The result of immunofluorescence staining showed that the average fluorescence intensity of cleaved-caspase 3 was higher than that of the control (Figure 2F). The results of flow cytometry showed that Cu^2+^ significantly increased the apoptosis rate in SH-SY5Y cells, especially in late apoptosis (Figure 2G), which was consistent with the Western blot results.

### 3.3. Cu^2+^ Induced Autophagosome Accumulation and Autophagic Flux Blockage in SH-SY5Y Cells

**Figure 3:**
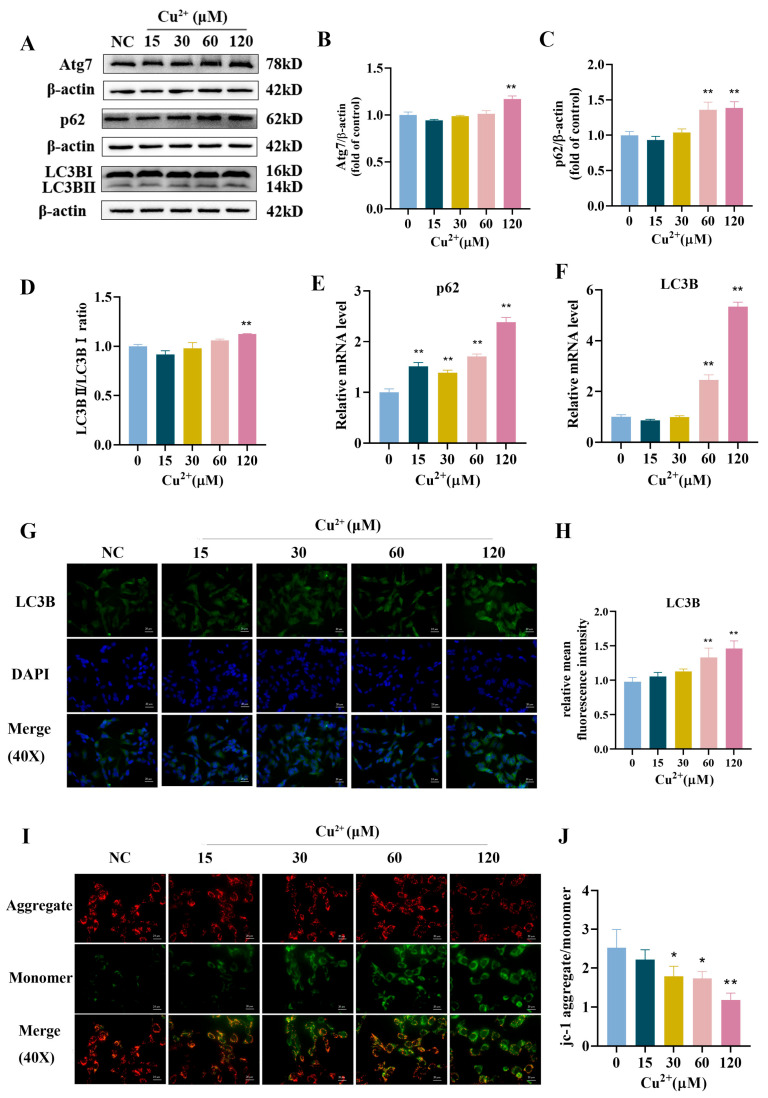
Cu^2+^ induced autophagic flux blockage in SH-SY5Y cells. (**A**) The protein expression levels of Atg7, p62 and LC3BII/LC3BI. (**B**–**D**) Quantitative analysis for protein expression of Atg7, p62, and LC3BII/LC3BI. (**E**,**F**) Quantitative analysis for mRNA expression of LC3B and p62. (**G**,**H**) The immunostaining detection of LC3B of SH-SY5Y cells (scale bar = 20 μm). (**I**,**J**) Typical images of SH-SY5Y cells stained by the JC-1 probe after Cu^2+^ treatment for 48 h (scale bar = 20 μm). * *p* < 0.05 and ** *p* < 0.01.

We evaluated the role of Cu^2+^ in autophagic flux by measuring the indicators of autophagy, LC3B and p62. The results of the Western blot indicated that Cu^2+^ dose-dependently increased the protein levels of Atg7, p62 and LC3BII/LC3BI in SH-SY5Y cells, with a noticeable increase in the high-dose group (Figure 3A–D, *p* < 0.01). The results of qPCR showed that Cu^2+^ significantly increased the gene expression levels of LC3B and p62 (Figure 3E,F). Immunofluorescence staining showed that expression of LC3B was significantly upregulated in the 60 μM and 120 μM Cu^2+^ groups in a dose-dependent manner (Figure 3G,H). Besides, the results of JC-1 exhibited that Cu^2+^ provoked a decrease in the ratio of JC-1 aggregate/monomer (Figure 3I,J). The results showed that copper induced autophagosome accumulation and autophagic flux blockage in SH-SY5Y cells, as well as cellular mitochondrial damage, and further confirmed that Cu^2+^ induced apoptosis in SH-SY5Y cells.

### 3.4. Cu^2+^-Induced Wnt Signaling Pathway Declined in SH-SY5Y Cells

**Figure 4:**
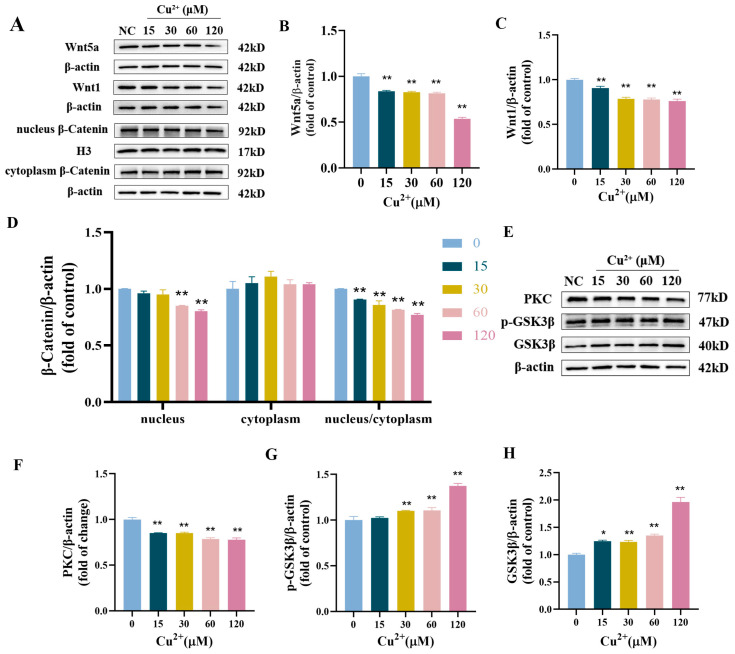
Cu^2+^ repressed the Wnt signaling pathway in SH-SY5Y cells. (**A**) The protein expression levels of Wnt1, Wnt5a and β-catenin. (**B**–**D**) Quantitative analysis for protein expression of Wnt1, Wnt5a and β-catenin. (**E**) The protein expression levels of PKC, p-GSK3β, and GSK3β. (**F**–**H**) Quantitative analysis for protein expression of PKC, p-GSK3β and GSK3β. * *p* < 0.05 and ** *p* < 0.01.

After 48 h of Cu^2+^ exposure, the contents of Wnt1, Wnt5a, and nucleus/cytoplasm β-catenin significantly decreased with the increase in Cu^2+^ levels (Figure 4A–D). We further detected the relative expression of key related molecules (GSK3β, p-GSK3β, and PKC). The results indicated that copper exposure significantly downregulated the expression of PKC, while it upregulated the expression of GSK3β and p-GSK3β (Figure 4E–H). The results revealed that copper exposure could, to some extent, repress Wnt signaling in SH-SY5Y cells.

### 3.5. Cu^2+^ Regulated the PI3K/Akt/mTOR Signaling Pathway

**Figure 5:**
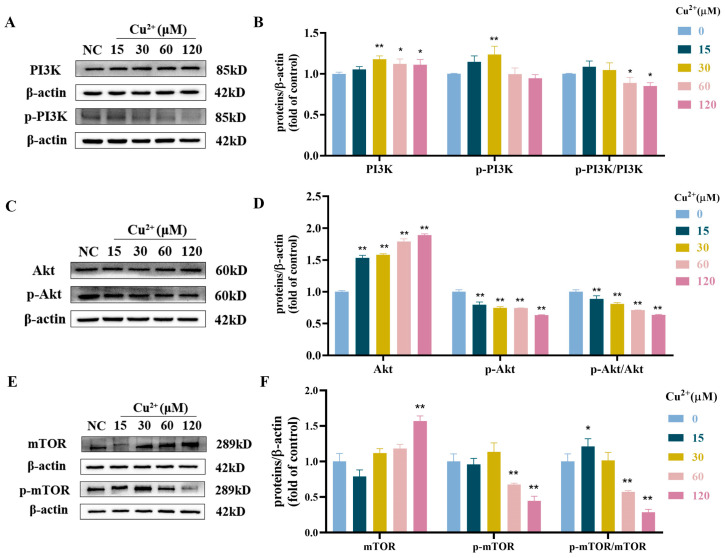
Cu^2+^ inhibited the PI3K/Akt/mTOR pathway. (**A**) The protein expression levels of PI3K, p-PI3K and p-PI3K/PI3K. (**B**) Quantitative analysis for protein expression of PI3K, p-PI3K and p-PI3K/PI3K. (**C**) The protein expression levels of Akt, p-Akt and p-Akt/Akt. (**D**) Quantitative analysis for protein expression of Akt, p-Akt and p-Akt/Akt. (**E**) The protein expression levels of mTOR, p-mTOR and p-mTOR/mTOR. (**F**) Quantitative analysis for protein expression of mTOR, p-mTOR and p-mTOR/mTOR. * *p* < 0.05 and ** *p* < 0.01.

The PI3K/Akt/mTOR signaling pathway is prominent for negative regulation of autophagy [41]. As shown in Figure 5A,B, the protein expression of p-PI3K/PI3K was decreased with copper treatment. PI3K and p-PI3K expression levels were increased after 30 μM Cu^2+^ treatment; however, the ratio of p-PI3K/PI3K had no statistical difference. Besides, the phosphorylation ratio of Akt and mTOR was also dose-dependently decreased after Cu^2+^ treatment (Figure 5C–F). The results showed that Cu^2+^ exposure inhibited the PI3K/Akt/mTOR pathway.

### 3.6. Rapa Rescued Apoptosis and Decreased Differentiation Capacity Induced by Cu^2+^ in SH-SY5Y Cells

**Figure 6:**
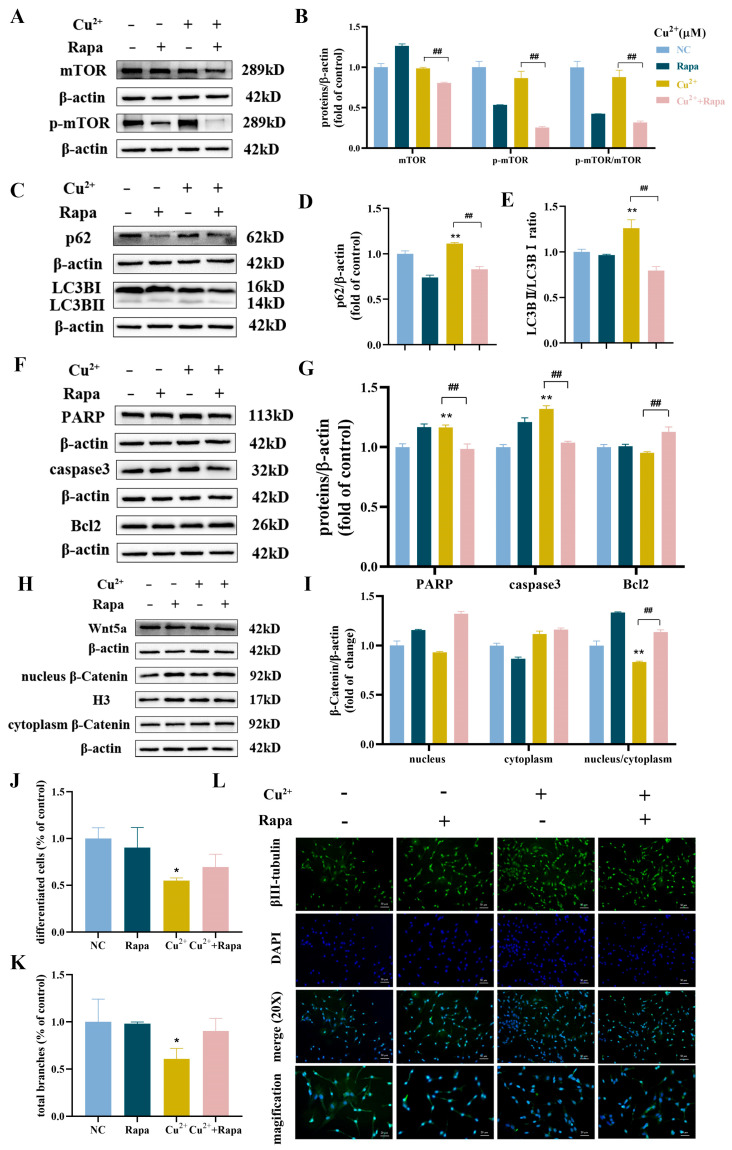
Rapa rescued apoptosis and decreased the differentiation capacity induced by Cu^2+^ in SH-SY5Y cells. (**A**) The protein expression levels and (**B**) quantitative analysis of mTOR and p-mTOR after Rapa pretreatment. (**C**) The protein expression levels and (**D**,**E**) quantitative analysis of p62 and LC3BII/LC3BI and after Rapa pretreatment. (**F**,**G**) The protein expression levels and quantitative analysis of PARP, caspase 3 and Bcl2 after Rapa pretreatment. (**H**,**I**) The protein expression levels and quantitative analysis of Wnt5a and β-catenin after Rapa pretreatment. (**J**) The proportions of differentiated cells after Rapa pretreatment. (**K**) The proportions of total branches of SH-SY5Y cells after Rapa pretreatment. (**L**) The immunostaining of βIII-tubulin of SH-SY5Y cells after Rapa pretreatment (scale bar-merge: 50 μm; magnification: 20 μm). (The hash symbol stands for the difference between the Rapa + 120 μM Cu^2+^ group and the 120 μM Cu^2+^-treated group alone: * *p* < 0.05, ** *p* < 0.01 and ^##^ *p* < 0.01).

Rapa is a specific inhibitor of the mTOR protein and an activator of autophagy [42]. To investigate the relationship between Cu^2+^-induced autophagy and differentiation capacity, the cells were pretreated with Rapa. As shown in Figure 6A,B, the protein expressions of p-mTOR and p-mTOR/mTOR were noticeably decreased after pretreatment with Rapa, compared to the 120 μM Cu^2+^-treated group alone. The protein expression levels of LC3BII/LC3BI, p62, PARP and caspase 3 were decreased, while Bcl2 was increased (Figure 6C–G). Meanwhile, the protein expression of Wnt5a and nuclear translocation of β-catenin were significantly increased after Rapa pretreatment (Figure 6H,I). On the other hand, compared to the 120 μM Cu^2+^-treated group alone, the proportions of differentiated cells and total branches were increased after Rapa pretreatment (Figure S2). Compared with the control group, the proportions of differentiated cells were 90%, 55% and 70% in the Rapa, Cu^2+^ and Cu^2+^ + Rapa groups (Figure 6J), and the proportions of total branches were 98%, 60% and 90% (control = 100%; Figure 6K). Meanwhile, the immunostaining of βIII-tubulin of SH-SY5Y cells showed that co-treatment with Rapa increased the expression of βIII-tubulin, alleviated the shorter neurites and less branches compared with the 120 μM Cu^2+^ group (Figure 6L). The results showed that Rapa alleviated autophagic degradation blockage induced by Cu^2+^, reduced accumulation of autophagosomes, reduced apoptosis, and enhanced cell differentiation in SH-SY5Y cells.

### 3.7. CQ Exacerbated Apoptosis and Decreased Differentiation Capacity Induced by Cu^2+^ in SH-SY5Y Cells

**Figure 7:**
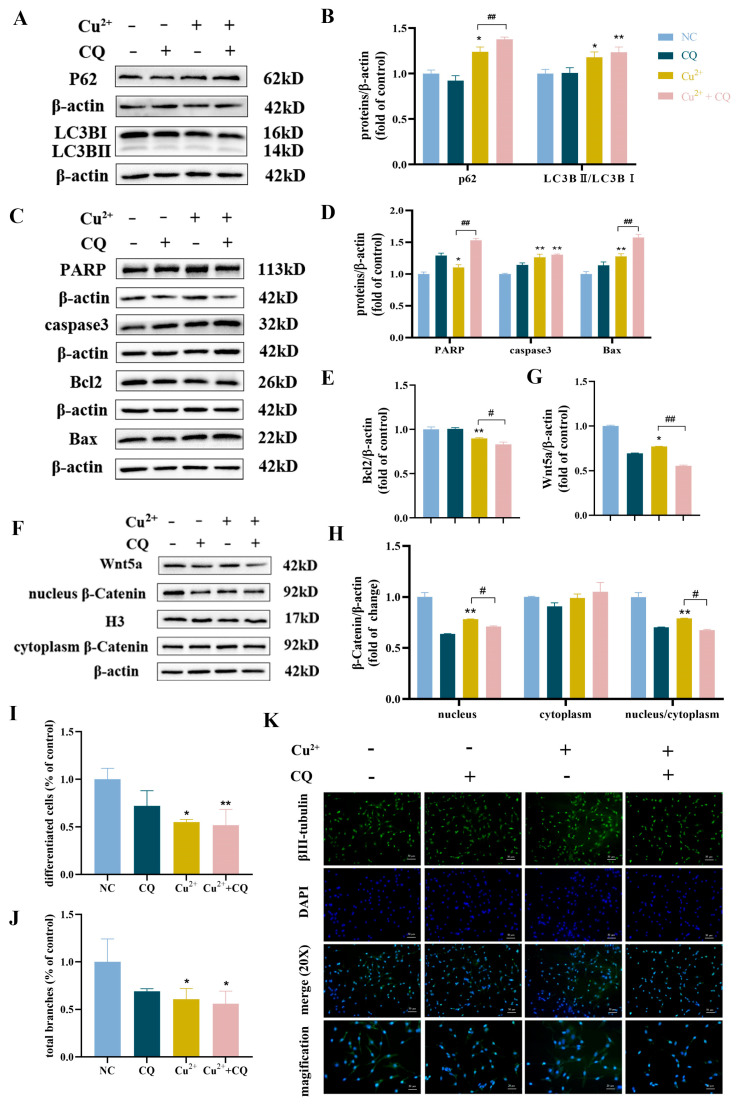
CQ exacerbated apoptosis and decreased the differentiation capacity induced by Cu^2+^ in SH-SY5Y cells. (**A**,**B**) The protein expression levels and quantitative analysis of p62 and LC3BII/LC3BI after CQ pretreatment. (**C**–**E**) The protein expression levels and quantitative analysis of PARP, caspase 3, Bax and Bcl2 after CQ pretreatment. (**F**–**H**) The protein expressions of Wnt5a, nucleus β-catenin and cytoplasm β-catenin were assessed by Western blot after CQ pretreatment. (**I**) The proportions of differentiated cells after CQ pretreatment. (**J**) The proportions of total branches of SH-SY5Y cells after CQ pretreatment. (**K**) The immunostaining of βIII-tubulin of SH-SY5Y cells after CQ pretreatment (scale bar-merge: 50 μm; magnification: 20 μm). * *p* < 0.05, ** *p* < 0.01, ^#^ *p* < 0.05, and ^##^ *p* < 0.01.

To further explore the relationship between the Cu^2+^-induced autophagy and differentiation capacity decrease in SH-SY5Y cells, the cells were pretreated with CQ. CQ is an inhibitor of autophagic flux by reducing autophagosome–lysosome fusion, inhibiting degradation blockage [43]. The results found that the protein expression levels of LC3BII/LC3BI and p62 were increased after CQ pretreatment compared to the 120 μM Cu^2+^-treated group alone (Figure 7A,B). The protein expression levels of PARP, caspase 3, and Bax were increased, while Bcl2 was decreased (Figure 7C–E). Meanwhile, the protein expression levels of Wnt5a and nucleus/cytoplasm β-catenin significantly decreased after CQ pretreatment (Figure 7F–H). On the other hand, the proportions of differentiated cells and total branches were decreased after CQ pretreatment: the proportions of differentiated cells were 72%, 55% and 52% and the proportions of total branches were 69%, 61% and 56% in the CQ, Cu^2+^ and Cu^2+^ + CQ groups (Figure 7I,J). The immunostaining of βIII-tubulin of SH-SY5Y cells showed that co-treatment with CQ caused decreased expression of βIII-tubulin, shorter neurites, and fewer branches compared with the 120 μM Cu^2+^ group (Figure 7K). These results indicated that CQ further aggravated autophagic flux blockage induced by Cu^2+^, increased accumulation of autophagosomes, induced apoptosis, and reduced cell differentiation.

## 4. Discussion

Copper has been widely considered to be one of the neurotoxins associated with several neurological diseases. Copper can cross the blood-brain barrier (BBB), as it is taken up by copper transporter 1 in neurons [44]. Although there is compelling evidence reporting that copper is harmful to neurodevelopment, further studies are necessary to explore the mechanisms leading to the potential damage to the nervous system. Neuronal growth, connectivity, and synapse formation are fundamental development processes of the nervous system [45]. The neurite outgrowth test is an influential component of the development of the nervous system and is applied to assess developmental neurotoxicity in vitro [46]. Thus, we established an in vitro model of Cu^2+^-exposed SH-SY5Y cells to investigate the effect of copper in developmental neurotoxicity. The results showed that the proportion of differentiated SH-SY5Y cells and the total number of branches were reduced after Cu^2+^ treatment. Besides, the immunofluorescence results of βIII-tubulin indicated that the neurons showed shorter and fewer branches. Altered expression of neurodevelopment-related genes could provide evidence for the potential of developmental neurotoxicity. βIII-tubulin, a differentiation and maturation neuronal marker, is a neuronal cytoskeletal protein that increases with the differentiation process [47]. Similar to this study, copper pyrithione decreased the total number branches of human SH-SY5Y/astrocytic co-cultured cells in [48]. These findings demonstrated that Cu might induce developmental neurotoxicity.

To gain insight into the mechanisms involved in copper-induced developmental neurotoxicity, we further investigated the Wnt signaling pathway. The Wnt signaling pathway plays a key role in neural development, and previous studies demonstrated that the signaling pathway plays prominent roles in neurogenesis, synapse formation, and neuronal maturation [49,50]. Wnt1 is a crucial component for the canonical Wnt signaling pathway, expressed in proliferating cells and increased during differentiation [51]. Wnt5a performs proper axon guidance and regulates glial progenitor cell proliferation and neuronal differentiation and specialization in the cerebellum [52]. Downregulation of the Wnt5a pathway could induce developmental neurotoxicity in rat pups, as evidenced by impaired spontaneous motor activity and cognitive function [53]. Since the Wnt signaling pathway plays crucial roles in neuronal processes, we evaluated whether Cu^2+^ would affect neuronal development through changes in Wnt signaling pathways. The results revealed that the protein expression levels of Wnt1, Wnt5a, PKC, and nuclear/cytoplasm β-catenin were decreased, while the protein expressions of GSK3β and p-GSK3β were increased. The nuclear translocation of β-catenin is essential for the regulation of the classical Wnt signaling pathway target genes. When Wnt signaling is off, β-catenin is degraded after being phosphorylated by the APC/Axin/GSK3β complex [18]. On the other hand, GSK3β plays a complicated role in neural development and adult neurogenesis. GSK3β was activated during ketamine-induced developmental neuroapoptosis in rats [54]. Activation of GSK3β upregulated the phosphorylation level of Tau protein, leading to decreased motor and exploratory abilities and impaired visuospatial perception in Wistar rats [55]. The results in this study confirmed that the Wnt signaling pathway was involved in Cu^2+^-induced developmental neurotoxicity. Similar to our results, environmental cadmium exposure significantly disturbed the expression levels of neuroinflammation factors and the Wnt signaling pathway, resulting in developmental neurotoxicity in zebrafish [56]. However, what are the potential mechanisms of copper-induced developmental neurotoxicity? This question is still very elusive.

We analyzed the expression of key proteins involved in apoptosis and autophagy. Apoptosis is thought to be an influential cause of neuronal loss. The process of mitochondria-mediated apoptosis is caused by an imbalance between the anti-apoptotic protein Bcl2 and the pro-apoptotic protein Bax [6]. Cu^2+^-induced translocation of Bcl2 protein from the cytoplasm to the mitochondria promotes the release of cytochrome C, stimulating the caspase cascade and apoptotic pathway [57]. Cleaved-caspase 3 leads to activation and cleavage of PARP1, promoting cell disassembly in SH-SY5Y cells exposed to copper [58]. In the present study, Cu^2+^ reduced MMP, accompanied with increased cell apoptosis, high expression levels of PARP, caspase 3, Bax, and cleaved-caspase 3, and low Bcl2 expression in SH-SY5Y cells. MMP is critical for the maintenance of cellular homeostasis, and decreased MMP leads to apoptosis [6]. This finding is consistent with that of Chakraborty, who found that 100 μM and 200 μM of CuSO_4_ caused alteration of MMP and promotion of apoptosis progression in SH-SY5Y cells [59]. In a word, the results suggested that Cu^2+^ induced apoptosis.

Autophagic flux is critical for maintaining intracellular homeostasis by forming autophagosomes that transport misfolded and aggregated proteins, defective organelles, and pathogens to lysosomes for degradation and recycling [60]. During the formation of autophagosomes, LC3I binds to phosphatidylethanolamine (PE) to form a lipoform of LC3, also known as LC3II, and the process is mediated by a ubiquitin-like reaction involving Atg7 and Atg3 [61]. LC3II is an autophagy marker protein essential for the autophagosomes’ formation, and the accumulation of autophagosomes is the result of increased formation or decreased degradation [62]. As our results showed, Cu^2+^ resulted in the accumulation of autophagosomes through detecting the upregulation of LC3II expression. p62 is an important substrate and receptor for the autophagy pathway, and accumulated p62 is always attributed to impaired autophagosome degradation [63]. Furthermore, the PI3K/Akt/mTOR pathway was detected in this study, which is a negative regulatory signal for autophagy. Copper induced autophagy through the PI3K/Akt/mTOR signaling pathway [64]. Consistent with the literature, this research found that the PI3K/Akt/mTOR signaling pathway was downregulated after Cu^2+^ treatment. The results indicated that Cu^2+^ induced both autophagosome accumulation and autophagic degradation blockage in SH-SY5Y cells.

Autophagy is involved in key events of neuronal development, such as regulating axon growth, dendritic complexity, spine pruning, and synapses’ formation [65]. Numerous studies suggested that autophagy deficiency is involved in the process of neural development. Pb-induced embryonic toxicity and impaired larval locomotor activity were excessive in zebrafish through inhibiting autophagosome formation, characterized by the lower expression of LC3II [66]. The 2,2′,4,4′-tetrabromodiphenyl ether (PBDE-47)-induced apoptosis, memory deficits, and developmental neurotoxicity in rats were associated with the accumulation of autophagic vesicles and increased levels of LC3II and p62 [67]. However, the developmental neurotoxicity in the above studies was rescued by Rapa, and autophagy protected neurodevelopment from environmental toxicants. Interestingly, the activation of autophagy damaged learning and memory abilities in rat hippocampus neurons [68]. Sevoflurane caused developmental neurotoxicity via increased autophagy, which was indicated by the increased level of LC3II, while there was a decreased expression of p62 in rat hippocampus neurons [69]. Differences in cell and rat specificity and dose sensitivity may account for the inconsistency.

To investigate the relationship between the copper-induced autophagy and differentiation capacity decrease of SH-SY5Y cells, the cells were treated with 120 μM of Cu^2+^ either in conjunction with or without Rapa and CQ. Our results showed that restoration of autophagic flux blockage and reduction of autophagosome accumulation induced by Cu^2+^ reverted the Wnt signaling pathway, as well as the proportion and total branches of differentiated SH-SY5Y cells. These results corroborated the findings of the previous work. Rapa promoted autophagosome-lysosome fusion, partly restored autophagic flux, and reduced apoptosis, eventually reducing developmental neurotoxicity induced by fluoride in SH-SY5Y cells [70]. Zhang et al. found that sodium fluoride impaired Sprague-Dawley offspring learning and memory capabilities by inducing autophagic flux blockage and apoptosis, which could be ameliorated by Rapa [40]. Treatment with CQ increased malformation rates and decreased survival rates and neurodevelopment-related proteins in zebrafish embryos [71]. The results further confirmed that the copper-induced developmental neurotoxicity in SH-SY5Y cells might be induced by autophagic degradation blockage.

On the other hand, autophagy can avert and inhibit apoptosis, and the protein networks are complex and intersecting [72]. Generally, autophagy inhibits apoptosis and reduces cell injury through decreasing the activation of the caspase protein family related to apoptosis [73]. In some conditions, autophagy and autophagy-related proteins might induce apoptosis, thus aggravating cell damage. The inhibition of the formation of autophagosome-targeted Atg3 or Atg5 reduced apoptosis [74]. In contrast, the activation of caspase inhibited autophagy via autophagy-related proteins, including Atg3, Atg4, and Beclin-1 [75]. However, it has been demonstrated that apoptosis and dysregulation of autophagic flux contribute to cytotoxicity induction, and autophagic flux blockage exacerbates apoptosis [76]. That might be a result of the physical interaction of p62 with caspase 8, which promotes self-aggregation and activation of caspase 8 [77]. Indeed, compared with the 120 μM Cu^2+^ treatment alone, induction of autophagy alleviated, while inhibition of autophagy aggravated copper-induced apoptosis. These results suggested that autophagy might be a target for mitigating copper-induced developmental neurotoxicity.

This study indicated that Rapa rescued apoptosis and decreased the differentiation capacity induced by copper in SH-SY5Y cells through reducing autophagic flux blockage. The opposite results were observed after pretreatment with CQ, an inhibitor of autophagic flux that acts by reducing autophagosome-lysosome fusion. The results further confirmed that the Cu^2+^-induced developmental neurotoxicity might be induced by autophagic degradation blockage.

In general, we have shown that Cu^2+^ exposure induced apoptosis and decreased the differentiation of SH-SY5Y cells, and this association might be related to the Wnt signaling pathway downregulated by autophagic flux blockage. The interactions between copper-induced autophagic degradation blockage, apoptosis, and the Wnt signaling pathway are complicated, and the specific target needs to be further investigated and validated by in vivo experiments.

## 5. Conclusions

In conclusion, this study provided evidence that Cu^2+^ reduced the neurite growth of SH-SY5Y cells through the Wnt signaling pathway. Meanwhile, Cu^2+^ induced apoptosis and led to autophagosome accumulation and autophagic degradation blockage through the PI3K/Akt/mTOR signaling pathway. This study might offer new insights into the role of autophagic flux blockage in developmental neurotoxicity caused by copper.

## Data Availability

All data included in this study are available upon request by contacting the corresponding author.

## Supplementary Materials

The following supporting information can be downloaded at: https://www.mdpi.com/article/10.3390/toxics13010063/s1. Figure S1: The differentiated SH-SY5Y cells morphology after Cu^2+^ exposure for 48 h (scale bar = 100 μm). Figure S2. The differentiated SH-SY5Y cells morphology after Rapa and CQ pretreatment in conjunction with or apart from 120 μM Cu^2+^ (scale bar = 100 μm).
