# Supplementary material for: The Role of Autophagy in Copper-Induced Apoptosis and Developmental Neurotoxicity in SH-SY5Y Cells
Source: Toxics. 2025 Jan 17;13(1):63. doi: 10.3390/toxics13010063 (PMC11769067; doi:10.3390/toxics13010063)
Supplement: Supplementary file 1 [file toxics-13-00063-s001.zip › toxics-3392253-supplementary.pdf]

The Role of Autophagy in Copper−Induced Apoptosis and Developmental Neurotoxicity in SH−SY5Y Cells

Lu Lu[1], Ying Zhang[1], Wei Shi[1], Qian Zhou[1], Zhuoqi Lai[1], Yuepu Pu[1], Lihong Yin[1 *]

[1] Key Laboratory of Environmental Medicine Engineering, Ministry of Education, School of Public Health, Southeast University, Nanjing, 210009, China

E-mails: 230229018@seu.edu.cn (L.L.); 101300315@seu.edu.cn (Z.Y); 230239085@seu.edu.cn (S.W.); 230229470@seu.edu.cn (Z.Q.); 220223707@seu.edu.cn (L.Z.); yppu@seu.edu.cn (Y.P.);

[*]Corresponding author: Lihong Yin;

87 Dingjiaqiao Street, Nanjing 210009, China

Tel.: +86-025-8327-2583;

Fax.: +86-025-8327-2583；

Email: lhyin@seu.edu.cn (L.Y.)

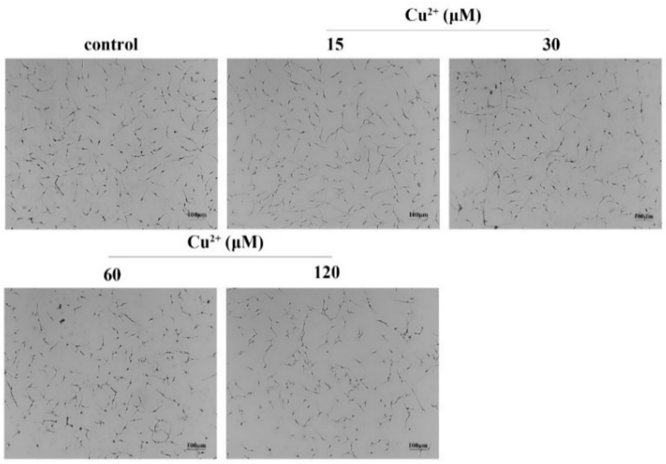

Figure S1. The differentiated SH−SY5Y cells morphology after Cu^{2+} exposure for 48 h (scale bar = 100 μm).

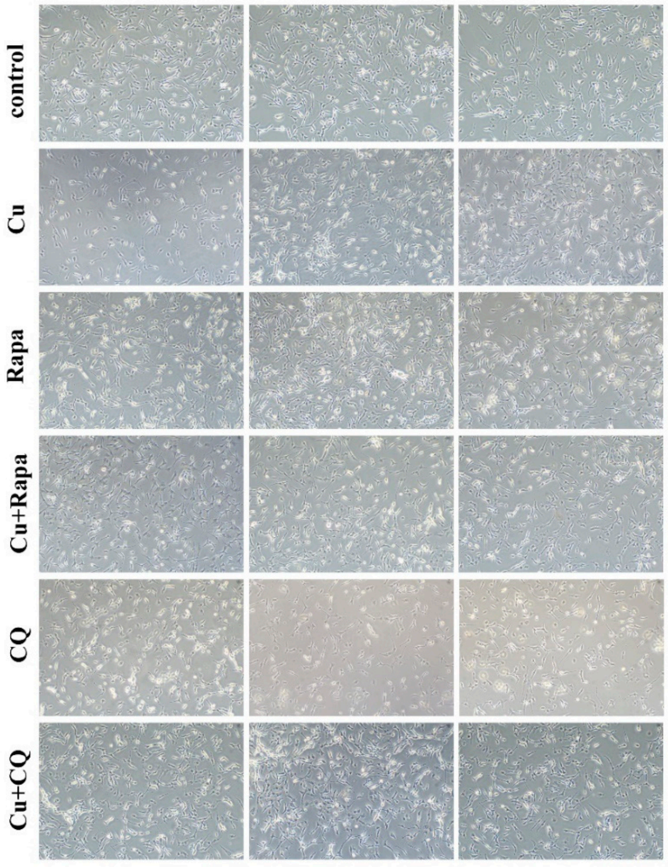

Figure S2. The differentiated SH−SY5Y cells morphology after Rapa and CQ pretreatment in conjunction with or apart from 120 μM Cu^{2+}. (scale bar = 100 μm).
